# Supplementary material for: Local circuit allowing hypothalamic control of hippocampal area CA2 activity and consequences for CA1
Source: eLife. 2021 May 18;10:e63352. doi: 10.7554/eLife.63352 (PMC8154026; doi:10.7554/eLife.63352)
Supplement: Figure 7—figure supplement 1—source data 1. [file elife-63352-fig7-figsupp1-data1.docx]

**Figure 7 – source data 1. Statistical comparisons related to Figure 7 figure supplement 1.**

| Measurement | Conditions | Factors | 2-way ANOVA p-values |
| --- | --- | --- | --- |
| amplitude | EPSC amplitude +/- CCh  (n = 13) | treatment | 0.00171693 |
|  |  | pulse # | 0.00286193 |
|  |  | treatment x pulse # | 0.0521822 |
|  | IPSC amplitude +/- CCh  (n = 13) | treatment | 0.413564 |
|  |  | pulse # | 0.0247487 |
|  |  | treatment x pulse # | 0.316489 |
|  | PSC amplitude in ACSF  (n = 13) | holding level | 0.0121691 |
|  |  | pulse # | 0.0115431 |
|  |  | holding level x pulse # | 0.391097 |
|  | PSC amplitude in CCh  (n = 13) | holding level | 2.85112E-11 |
|  |  | pulse # | 0.189593 |
|  |  | holding level x pulse # | 0.55014 |
| Pn/P1 ratio | EPSC Pn/P1 ratio +/- CCh  (n = 13) | treatment | 1.05342E-10 |
|  |  | pulse # | 9.99201E-16 |
|  |  | treatment x pulse # | 0.0110396 |
|  | IPSC Pn/P1 ratio +/- CCh  (n = 13) | treatment | 0.000184435 |
|  |  | pulse # | 0.00209369 |
|  |  | treatment x pulse # | 0.297716 |
|  | PSC Pn/P1 ratio in ACSF  (n = 13) | holding level | 0.325751 |
|  |  | pulse # | 2.08101E-08 |
|  |  | holding level x pulse # | 0.941122 |
|  | PSC Pn/P1 ratio in CCh  (n = 13) | holding level | 0.0948351 |
|  |  | pulse # | 3.07005E-05 |
|  |  | holding level x pulse # | 0.889375 |
| E/I ratio | PSC E/I ratio +/- CCh  (n = 13) | treatment | 7.61696E-06 |
|  |  | pulse # | 0.99245 |
|  |  | treatment x pulse # | 0.982047 |
